# Antifungal susceptibility and phenotypic virulence markers of *Candida* species isolated from Nepal

**DOI:** 10.1186/s13104-017-2852-x

**Published:** 2017-11-02

**Authors:** Supram Hosuru Subramanya, Bharat Prasad Baral, Nawal Kishor Sharan, Niranjan Nayak, Yang Metok, Brijesh Sathian, Indira Bairy, Shishir Gokhale

**Affiliations:** 1 Manipal College of Medical Sciences, Pokhara, Nepal; 2 Melaka Manipal Medical College, Manipal, India

**Keywords:** *Candida* species, Virulence factors, Antifungal susceptibility testing

## Abstract

**Objective:**

*Candida* species are part of the commensal microflora in many anatomical sites of the human body; however, breach in the integrity of the body part and impaired immunity of the host can lead to invasive candidiasis. A number of virulence determinants could contribute towards its pathogenicity. Thus we attempted to evaluate the in vitro expression of different virulence factors among clinical isolates of *Candida* species and assayed their susceptibility patterns against a range of antifungal agents.

**Result:**

Of the total of 71 isolates we obtained, 48 (67.6%) were *Candida albicans*, 11 (15.49%) *Candida tropicalis,* 09 (12.67%) *Candida glabrata* and 03 (4.22%) were *Candida krusei*. Proteinase, phospholipase and esterase production could be revealed amongst 43 (60.56%), 44 (61.97%) and 49 (69.01%) isolates respectively. None of the isolates showed DNAase activity. Fifty-five (77.39%) isolates were biofilm producers, and 53 (74.6%) exhibited high cell surface hydrophobicity.

**Electronic supplementary material:**

The online version of this article (10.1186/s13104-017-2852-x) contains supplementary material, which is available to authorized users.

## Introduction

*Candida* species are part of the commensal microflora in many anatomical sites of the human body [1]. If host immunity is compromised, or there is disruption in the skin or mucosal site where *Candida* remains as a commensal, there is always a chance for *Candida* to invade and cause a wide range of infections with significant morbidity and mortality [2, 3]. Though *Candida albicans* has been associated with most human infections, there has been increasing reports of infections due to non-albicans *Candida* species in the recent past [4, 5]. A number of virulence attributes such as biofilm formation, proteinase, esterase, phospholipase activities and drug resistance contributing towards the pathogenicity of *Candida* have been proposed. Thus our study was conducted to determine and compare in vitro production of virulence factors by *Candida* species and their antifungal susceptibility patterns. To the best of our knowledge, the present study is the first of its kind in Nepal.

## Main text

### Materials and methods

#### Candida isolates

A total of 71 *Candida* species isolated between 2014 and 2016 from various clinical samples (Additional file 1: Table S1) were studied. Organisms were identified by the standard laboratory techniques [6], and growth on HiChrome candida differential agar (Hi-Media, India).

#### Preparation of yeast suspension for enzymatic activity

A loopful of the culture was streaked onto Sabouraud’s dextrose agar (SDA) with chloramphenicol (0.05 g/l; HiMedia, India) and incubated at 37 °C for 24–48 h. Cells were harvested and suspended in sterile PBS and matched to 0.5 Mc Farland. The final suspension was adjusted to contain 2.5 × 10^6^ yeast cells/ml. The above inocula were used for all enzymatic studies, as well as for the biofilm activity. Qualities of all assays were checked using known positive and negative controls.

#### Biofilm formation

**Fig. 1:**
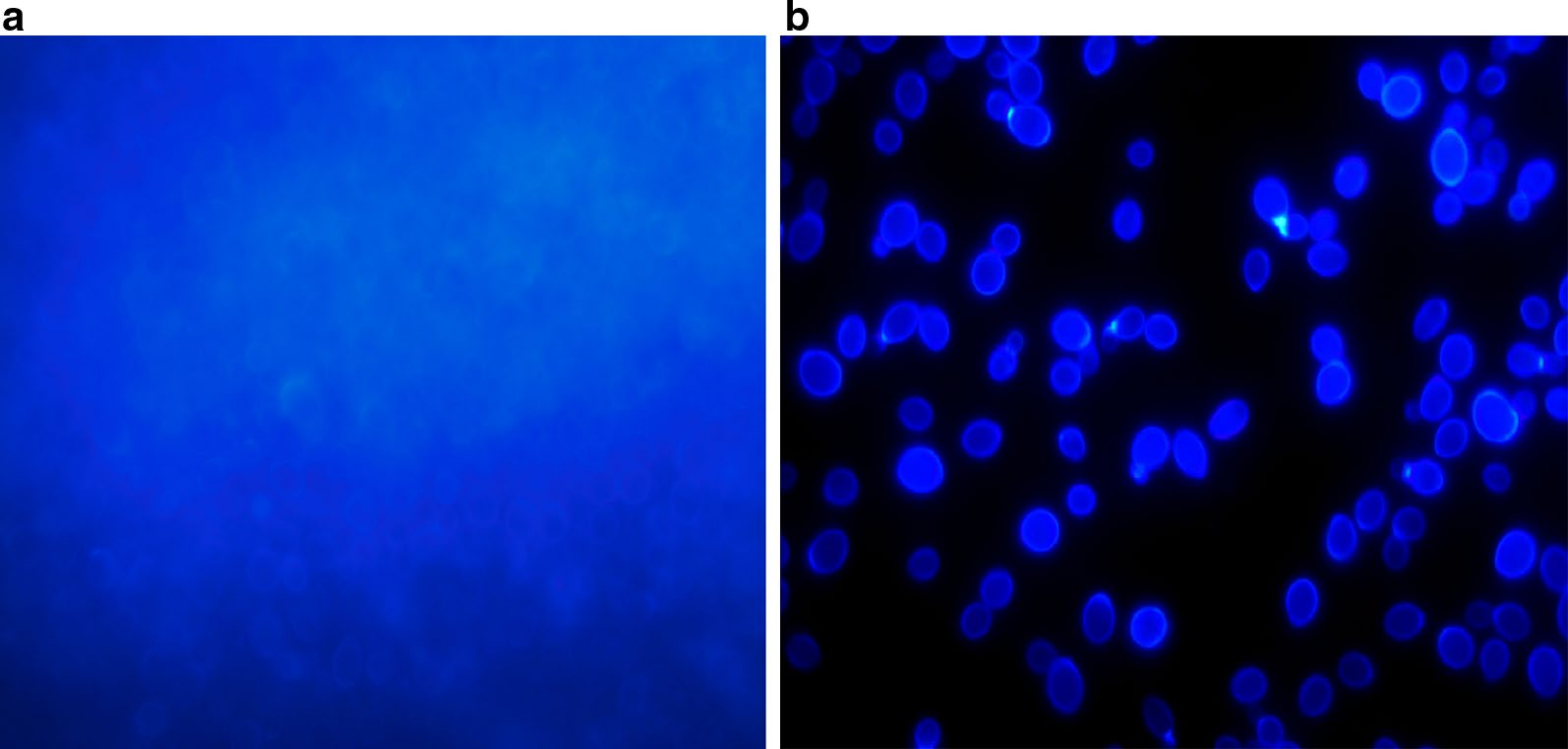
Demonstration of biomass of biofilm by fluorescent microscopy using calcofluor white stain: Calcofluor staining of biofilm produced in the glass slide showing (**a**) hazy appearance which was due to diffuse staining of the extracellular material (cell-wall-like polysaccharides). Blastospore communities were covered by the matrix (rarely seen) and (**b**) Planktonic cells of *Candida albicans* showing oval budding blastospores (magnification × 1000)

The method standardised by Malek et al. [6] was followed to develop biofilms in 96 well microtiter plates. Measurement of biofilm mass by quantitative method was performed using crystal violet for staining the biomass and metabolic activity of the biofilm cells was assessed colorimetrically based on reduction of sodium 39-[1-(phenylamino-carbonyl)-3,4-tetrazolium]-bis(4-methoxy-6-nitro)benzene sulfonic acid hydrate (XTT) as described elsewhere [7]. Biomass was also demonstrated by fluorescent microscopy with calcofluor white staining (Fig. 1). Known biofilm producer and non-biofilm producer *Candida* strains served as positive and negative controls respectively.

#### Proteinase and phospholipase activities

Proteinase activity was assessed by bovine serum albumin (BSA) agar based assay as described previously [8]. The presence of halo surrounding the growth representing proteinase activity was observed by staining with amido black. The egg yolk agar method as described earlier was employed for determining the phospholipase activity [9]. Pz (precipitation zone) values for both the tests were calculated according the parameters noted earlier [8, 9]. Known proteinase and phospholipase positive and negative *Candida* strains served as controls.

#### Esterase, deoxyribonuclease and cell surface hydrophobicity

Esterase activity was noted in Tween-80 agar as described previously [10] and test for cell surface hydrophobicity (CSH) was performed in accordance with the earlier devised technique [11]. DNase production was measured according to the standard protocol [11], using ATCC 25923 standard strain of *Staphylococcus aureus* as positive control. Known Esterase and CSH positive and negative *Candida* strains served as controls.

#### Antifungal susceptibility testing

All 71 isolates were subjected to antifungal susceptibility testing against amphotericin B, voriconazole, fluconazole and caspofungin by microbroth dilution method based on the Clinical and Laboratory Standards Institute M27-A3 standard [12]. Tests were interpreted by visual method with the help of reading mirror after 24 h of incubation at 37 °C. *Candida parapsilosis* ATCC 95142 and *C. albicans* ATCC 90028 were used as controls. Antifungal compounds were obtained as pure powders from the manufacturer, Sigma-Aldrich Laborchemikalien GmbH, Germany.

#### Statistical analysis

Descriptive statistics were used for analyzing the data entered in Microsoft Excel 2010 by Statistical Analysis System (SAS) and Origin Pro 2016. MIC values of different antifungal agents against *C. albicans* and non albicans *Candida* were expressed in terms of range, median and geometric mean. Variations among MIC values of antifungal agents against biofilm producing and non-biofilm producing *Candida albicans* and non-albicans *Candida* species were assessed by using minimum, maximum, median, and 90th percentile and box plot.

### Results

Out of a total of 71 *Candida* isolates, 48 (67.6%) were *C. albicans*; 11 (15.5%) were *C. tropicalis*; 9 (12.7%) *C. glabrata* and 3 (4.2%) were *C. krusei.* As depicted in Table 1, proteinase, phospholipase and esterase activity could be detected amongst 43 (60.6%), 44 (62%), and 49 (69%) of the isolates respectively. None of the isolates produced DNase. CSH, was observed among 54 (76%) of the 71 isolates. As many as 55 (77.4%) out of the total 71 isolates were found to be biofilm producers as evidenced by metabolic activity and biomass production (Fig. 1). Majority, i.e. 40 (74.07%) of the 54 having high cell surface hydrophobicity produced biofilms. 36 (75%) out of 48 *C. albicans* strains produced proteinase in contrast to only 7 (30%) of the 23 non-albicans *Candida* species. Similarly, higher numbers of *C. albicans* strains were found to be phospholipase and esterase producers as compared to non albicans *Candida* (Table 1). Isolation rates of *C. albicans* from blood and indwelling devices were found to be much higher as compared to non albicans *Candida* species. Similarly biofilm production was seen among 84–100% of the blood and device isolates (Additional file 1: Table S1).

**Table 1:** Virulence factors found among different *Candida* species

	Pz value	*Candida albicans* (n = 48)	Non albicans *Candida* species		
			*Candida tropicalis* (n = 11)	*Candida glabrata* (n = 9)	*Candida krusei* (n = 3)
Proteinase test					
Strong	< 0.79	35	5	1	0
Mild	0.80–0.89	1	1	0	0
Weak	0.90–0.99	0	0	0	0
Negative	= 1	12	5	8	3
Phospholipase test					
Strong	< 0.79	17	2	1	0
Mild	0.80–0.89	8	0	0	0
Weak	0.90–0.99	10	3	2	1
Negative	= 1	13	6	6	2
Esterase test					
Strong	< 0.79	31	8	3	1
Mild	0.80–0.89	6	0	0	0
Weak	0.90–0.99	0	0	0	0
Negative	= 1	11	3	6	2
Biofilm production					
Positive	–	36	10	6	3
Negative	–	12	1	3	0
Cell surface hydrophobicity (%)					
Strong	> 20	35	10	7	2
Mild	10–19.99	9	1	2	1
Weak	0.1–9.99	4	0	0	0
Negative	< 0.1	0	0	0	0

Tables 2 and 3 denote the antifungal susceptibility patterns of the isolates. Isolates were classified as sensitive, intermediately sensitive and resistant to each antifungal agent in accordance with the break point criteria laid down by CLSI [10]. During data analysis, both sensitive and intermediately sensitive isolates were categorized as one group, i.e. sensitive. As high as 97.9% (47/48), 85.4% (41/48) and 77% (37/48) *C. albicans* isolates were sensitive to amphotericin B, caspofungin and voriconazole respectively. Overall, 95.7% (22/23) of non-*albicans* strains were found to be susceptible to amphotericin B and caspofungin. Amongst *C. tropicalis* all 11, i.e. 100% were sensitive to amphotericin B and caspofungin (Table 3). Fluconazole sensitivity of *C. albicans*, *C. tropicalis* and *C. krusei* ranged between 33.3 and 52%. A total of 82.6% (19/23) of the non-albicans *Candida* were sensitive to voriconazole, only 56.5% (13/23) were sensitive to fluconazole.

**Table 2:** In vitro antifungal susceptibility profile of various *Candida* species

CLSI breakpoints (μg/ml)	Amphotericin B			Caspofungin			Fluconazole			Voriconazole		
	S	I	R	S	I	R	S	I	R	S	I	R
	≤ 4	8–16	≥ 32	≤ 0.25	0.5	≥ 1	≤ 2	4	≥ 8	≤ 0.12	0.25–0.5	≥ 1
*C. albicans,* n = 48 (%)	47 (97.9)	0 (0)	1 (2.1)	41 (85.4)	4 (8.3)	3 (6.3)	11 (23)	14 (29)	23 (48)	37 (77)	11 (23)	0 (0)
*C. tropicalis,* n = 11 (%)	0 (0)	11 (100)	0 (0)	11 (100)	0 0	0 0	4 (36.4)	1 (9)	6 (54.6)	0 0	9 (81.8)	0 (18.2)
*C. glabrata,* n = 9 (%)	0 (0)	9 (100)	0 (0)	9 (100)	0 0	0 0	6 (66.7)	1 (11.1)	2 (22.2)	0 0	8 (88.9)	1 (11.1)
*C. krusei,* n = 3 (%)	0 0	2 (66.7)	1 (33.3)	2 (66.7)	1 (33.3)	0 (0)	1 (33.3)	0 (0)	2 (66.7)	0 0	2 (66.7)	1 (33.3)

**Table 3:** MIC values of different antifungal agents against *C. albicans* and non albicans *Candida*

*Candida* isolates	Fluconazole MIC			Amphotericin B MIC			Voriconazole MIC			Caspofungin MIC		
	Range	Median	GM	Range	Median	GM	Range	Median	GM	Range	Median	GM
*C. albicans*	0.125–64	4	5.039	0.031–32	0.25	0.3	0.031–0.5	0.125	0.1	0.031–2	0.0625	0.068
Non albicans *Candida*	0.0313–64	4	3.718	0.0625–32	0.5	0.417	0.031–0.5	0.0625	0.077	0.031–0.5	0.0625	0.0625

Median MICs and geometric mean MIC (GMM) values of fluconazole were found much higher as compared to those for amphotericin B, caspofungin and voriconazole both for *C. albicans* and non albicans *Candida* (Table 3). While the median MICs and GMM values were found to be the lowest with respect to caspofungin in both *C. albicans* (0.00625 and 0.068 μg/ml) and non-albicans *Candida* (0.0625 and 0.0628 μg/ml) species, those for voriconazole were 0.125 and 0.1 μg/ml respectively against *C. albicans* and 0.0625 and 0.077 μg/ml respectively against non-albicans *Candida.*

**Fig. 2:**
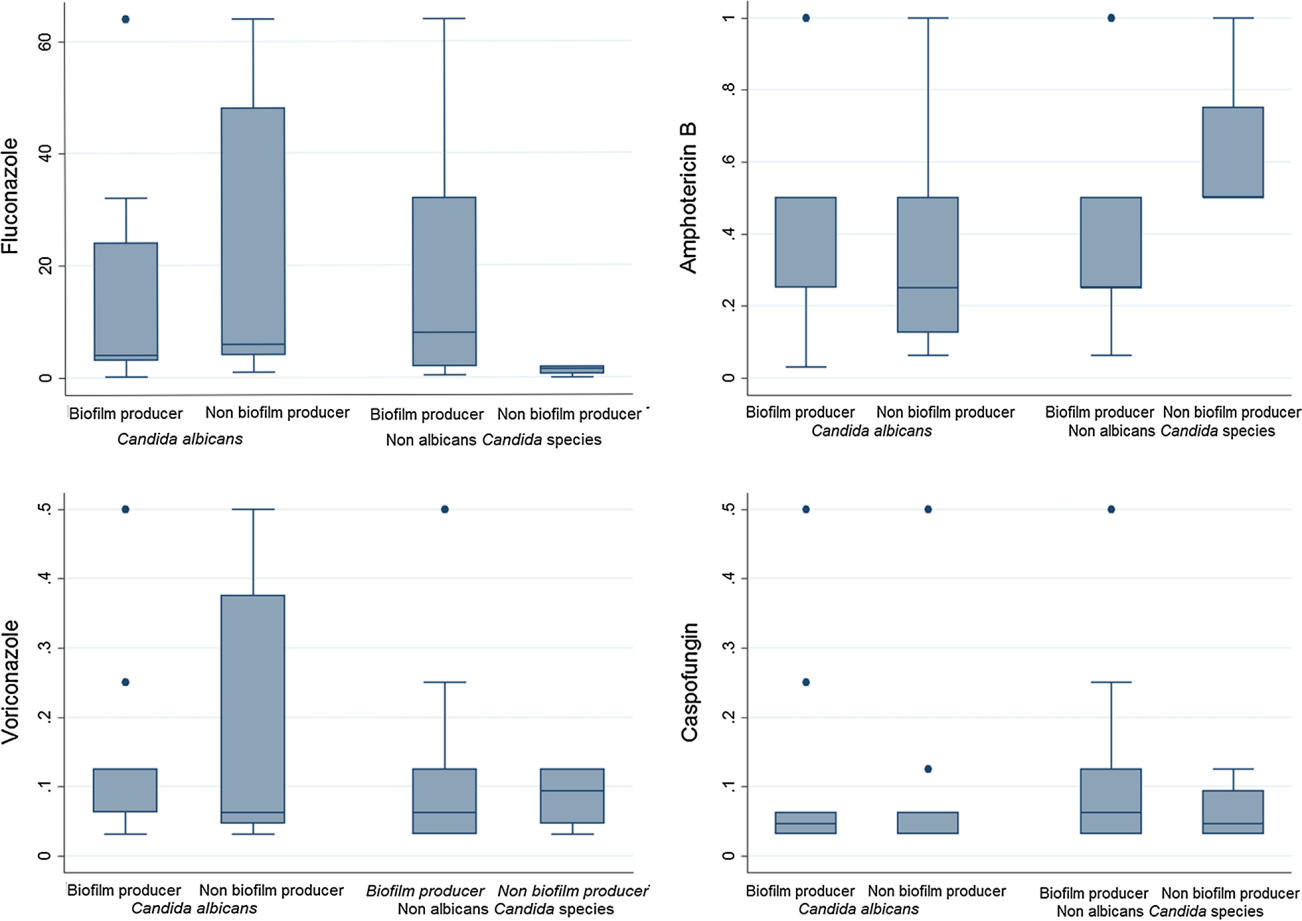
A box plot representation of MIC values (µg/ml) for different antifungal agents shown by biofilm producing and non-biofilm producing *Candida albicans* and non-albicans *Candida* species. *C. albicans* (n = 48) and biofilm producers (n = 36); fluconazole MIC values: maximum—64; minimum—1; median—4; 90th percentile—64. Amphotericin B MIC values: maximum—1; minimum—0.3; median—0.5; 90th percentile—0.65. Voriconazole MIC values: maximum—0.5; minimum—0.03; median—0.125; 90th percentile—0.25. Caspofungin MIC values: maximum—2; minimum—0.03; median—0.046; 90th percentile—0.50. *C. albicans* non-biofilm producers (n = 12): fluconazole MIC values: maximum—64; minimum—1; median—6; 90th percentile—64. Amphotericin B MIC values: maximum—32; minimum—0.06; median—0.25; 90th percentile—22.5. Voriconazole MIC values: maximum—0.5; minimum—0.03; median—0.062; 90th percentile—0.50. Caspofungin MIC values: maximum—1; minimum—0.03; median—0.062; 90th percentile—0.737. Non albicans *Candida* (n = 23) and biofilm producers (n = 19); fluconazole MIC values: maximum—64; minimum—0.50; median—8; 90th percentile—64. Amphotericin B MIC values: maximum—32; minimum—0.062; median—0.25; 90th percentile—1. Voriconazole MIC values: maximum—0.5; minimum—0.03; median—0.062; 90th percentile—0.50. Caspofungin MIC values: maximum—0.50; minimum—0.031; median—0.062; 90th percentile—0.25. Non albicans *Candida* non-biofilm producers (n = 4): Fluconazole MIC values: maximum—2; minimum—0.03; median—1.5. Amphotericin B MIC values: maximum—1; minimum—0.5; median—0.5. Voriconazole MIC values: maximum—0.12; minimum—0.03; median—0.093. Caspofungin MIC values: maximum—0.125; minimum—0.031; median—0.046

Based upon the median MIC data, we determined the number of strains showing high MIC values (higher than the median MIC) and those exhibiting low MIC values (lower than the median MIC), in order to see if there was any correlation between biofilm production and drug resistance. A significant difference could be noted amongst the non-albicans *Candida*, against amphotericin B, fluconazole and caspofungin. As depicted in Fig. 2, a large number of the non-biofilm producing *C. albicans* strains showed high MIC values against amphotericin B, fluconazole and voriconazole. Similarly, non-albicans *Candida* that were non-biofilm producers exhibited moderately higher MICs against amphotericin B.

### Discussion

Many invasive *Candida* infections are attributable to some of the potential virulence factors of the organism such as proteinase, phospholipase and esterase. Majority (72–77%) of the *C. albicans* strains in this study were capable of producing proteinase, phospholipase and esterase. A high rate of phospholipase (94.7%) and a moderately high rate of proteinase (73.7%) production amongst *C. albicans* clinical isolates were reported earlier [13–15]. Proteinase as a major virulence determinant of both *C. albicans* and non albicans *Candida* in invasive infections was documented earlier [16]. Gokee et al. [17] detected proteinase in 89.7% of *C. albicans* isolates, and only in 25.8% of the non-albicans isolates. Inci et al. [18] reported that 95% *C. albicans* and 24% non-albicans *Candida* were proteinase producers. We noted proteinase production among 75% of our *C. albicans* isolates and only 30% of the non-albicans isolates.

The role of esterase in the pathogenesis of invasive candidiasis is debatable [14–16]. However, earlier studies [18] demonstrated that both *C. albicans* and non albicans *Candida* species showed esterase activity. In our study, esterase was detected amongst 77% of *C. albicans* isolates as compared to 52% non-albicans isolates, difference being marginally higher among the *C. albicans* isolates. Tellapragada et al. [19] found no significant difference in the esterase activities among invasive and non-invasive *Candida*. They did not, however, compare this observation between *C. albicans* and non albicans isolates.

In the present study, resistance rates for the azoles were substantially higher as compared to amphotericin B or caspofungin, especially in *C. tropicalis* (Table 2). Additionally MIC ranges, median MICs and geometric mean titres were higher for fluconazole both for *C. albicans* and non-albicans *Candida* (Table 3). These findings were similar to those reported by others [20], who proposed that azole resistance in *Candida* was of concern because azoles like fluconazole happened to be the most common antifungal agent used for the treatment and prophylaxis of candidiasis in organ transplant recipients. In yet another study [5], nosocomial isolates of *C. albicans* were shown to have far lower sensitivity rates towards fluconazole. Seneviratne et al. [21] very recently reported that 31.7% of the *Candida* isolated from blood were resistant to fluconazole.

Apart from antifungal drug resistance in *Candida*, another major virulence attribute of this organism is production of biofilm that could lead to treatment failure and recurrence of infection. Tallaprageda et al. [19] noted high rate of biofilm production among the *Candida* isolates from blood stream and other invasive infections. Hassan et al. [22] found that significantly larger number of *C. albicans* isolates were biofilm producers as compared to the non-albicans *Candida*. While the exact reason for the higher rate of biofilm production among *C. albicans* was ill-understood, scanning electron microscopy studies of complex biofilm architectures attributed the integrity and strength of these biofilms to the higher number of hyphal elements produced by *C. albicans* than *C. tropicalis* and *C. parapsilosis*. The latter two species formed biofilms of lesser strength and the biofilm formed by these two species primarily consisted of micro colony aggregates of yeast cells [19]. In another recent investigation, Sariguzel et al. [11] detected biofilm among 33% of non albicans *Candida* as compared to 25% of *C. albicans*. We also observed a comparatively higher rate of biofilm production among non albicans *Candida* as opposed to *C. albicans* (83% vs. 75%; Table 1). Notwithstanding the aforementioned variability in the rate of biofilm production among different *Candida* species, it is noteworthy that such high degree of biofilm forming ability among clinical *Candida* isolates reflects the potential of these organisms to cause invasive disease [4, 22]. Thus, biofilm production could be a classic prototypical phenotypic marker of pathogenicity of a distinct population of *Candida*, differentiating these from mere commensals [1, 2, 23].

We observed that non-biofilm producing *C. albicans* and non albicans *Candida* showed high MICs towards fluconazole, amphotericin B and voriconazole. This correlation, however, could not be detected among *C. albicans* isolates (Fig. 2). Unlike in bacterial pathogens [24], studies involving correlation between biofilm production and multidrug resistance among *Candida* are scanty [20, 25–27]. Our study, however, highlighted that majority of the non albicans *Candida* strains that were biofilm producers had shown high MICs towards fluconazole (Fig. 2).

### Conclusion

Non-albicans *Candida* species are emerging as potential threats to cause invasive disease and posing a therapeutic challenge. Detection of high rate of biofilm activities among non-albicans *Candida* species along with high level of fluconazole resistance warrant wider surveillance of *Candida* isolates in order to clearly define the exact role of biofilms and drug resistance in invasive candidiasis.

### Limitations

Non-albicans *Candida* isolates in our study were few. Thus in order to hypothesise that more of non-albicans *Candida* were capable of forming biofilms as compared to *C. albicans*, further studies, including higher numbers, would be required. Testing of hypothesis of inferential statistics was not applicable for this study because of the inadequate sample size.

## Additional file

**Additional file 1: Table S1.** Isolation rates and percentage isolation of *C. albicans* vs. non-albicans *Candida* including their biofilm producing abilities in various clinical specimens.

## Abbreviations

ATCC: American Type Culture Collection
BSA: bovine serum albumin
CFG: caspofungin
CFU: colony forming units
CLSI: Clinical and Laboratory Standards Institute
CSH: cell surface hydrophobicity
DNAse: deoxyribonuclease
MIC: minimum inhibitory concentration
PBS: phosphate buffered saline
SDA: sabouraud dextrose agar
Vs: versus
XTT: sodium 39-[1-(phenylamino-carbonyl)-3,4-tetrazolium]-bis(4-methoxy-6-nitro)benzene sulfonic acid hydrate
