# Supplementary material for: Antifungal susceptibility and phenotypic virulence markers of Candida species isolated from Nepal
Source: BMC Res Notes. 2017 Nov 2;10:543. doi: 10.1186/s13104-017-2852-x (PMC5669004; doi:10.1186/s13104-017-2852-x)
Supplement: Supplementary file 1 — Additional file 1: Table S1. Isolation rates and percentage isolation of C. albicans vs. non-albicans Candida including their biofilm producing abilities in various clinical specimens. [file 13104_2017_2852_MOESM1_ESM.docx]

**Supplementary file**

**Table S1: Isolation rates and percentage isolation of *C albicans* vs. non-albicans *Candida* including their biofilm producing abilities in various clinical specimens**

| **Isolate no.** | **Clinical specimen** | ***C. albicans***  ***48(***67.61%) | **Non*-*albicans *Candida*** 23(32.39%) | **Biofilm**  **55 (78**%) |
| --- | --- | --- | --- | --- |
| 1 | Blood (13) | 12(92.3%) | 1(7.69%) | 11(84.6% ) |
| 2 | Feces (5) | 3(60%) | 2(40%) | 2 (40% ) |
| 3 | Indwelling medical divices (15) | 13(86.6%) | 2(13.3%) | 15 (100%) |
| 4 | High vaginal swab (11) | 8(72.7%) | 3(27.2%) | 8 (72.7% ) |
| 5 | Respiratory specimens (endotracheal aspirate, sputum, BAL fluid) (13) | 6(46.1%) | 7(53.8%) | 10 (76.9% ) |
| 6 | Body fluids (plural fluid , peritoneal fluid, CSF) (5) | 3(60%) | 2(40%) | 3(60%) |
| 7 | Urine (9) | 3(33.3%) | 6(66.6%) | 6 (66.7% ) |
